# Primary Metabolism in Citrus Fruit as Affected by Its Unique Structure

**DOI:** 10.3389/fpls.2019.01167

**Published:** 2019-09-26

**Authors:** Avi Sadka, Lyudmila Shlizerman, Itzhak Kamara, Eduardo Blumwald

**Affiliations:** ^1^Department of Fruit Tree Sciences, Institute of Plant Sciences, Agricultural Research Organization, The Volcani Center, Rishon LeZion, Israel; ^2^Department of Plant Sciences, University of California, Davis, Davis, CA, United States

**Keywords:** acidity, amino acids, citrate, citrus fruit, sugars, primary metabolism, quality

## Abstract

Citrus is one of the world’s most important fruit crops, contributing essential nutrients, such as vitamin C and minerals, to the human diet. It is characterized by two important traits: first, its major edible part is composed of juice sacs, a unique structure among fruit, and second, relatively high levels of citric acid are accumulated in the vacuole of the juice sac cell. Although the major routes of primary metabolism are generally the same in citrus fruit and other plant systems, the fruit’s unique structural features challenge our understanding of carbon flow into the fruit and its movement through all of its parts. In fact, acid metabolism and accumulation have only been summarized in a few reviews. Here we present a comprehensive view of sugar, acid and amino acid metabolism and their connections within the fruit, all in relation to the fruit’s unique structure.

## Citrus Fruit Morphology — A Unique Structure That Determines Critical Aspects of Primary Metabolism

The citrus fruit, termed hesperidium, is a fleshy fruit which, like all berry-type fruit, is characterized by a thick and fleshy pericarp (Esau, 1966; Fahn, 1990). The pericarp is usually divided into three tissues: the exocarp, which is the outer skin, the mesocarp, which usually refers to the major fleshy, edible interior, and the endocarp, an internal tissue composed of one (as in tomato) or several cell layers. In true fruit, which develop from the ovary, these three tissues are part of the ovary wall.

**Figure 1:**
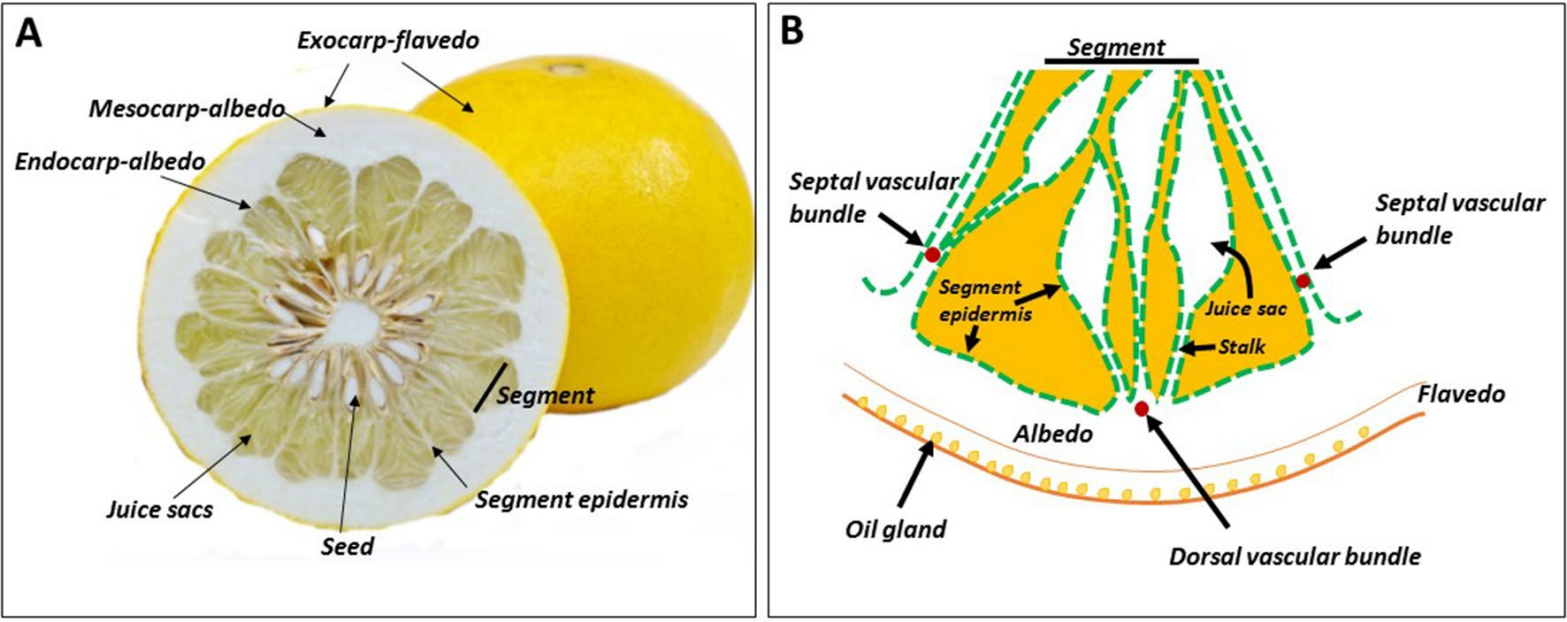
Citrus fruit morphology. Cross section of the fruit **(A)**, and schematic representation of a segment, including the peel tissues **(B)**. The segment epidermis also covers the juice sacs, and the three segment vascular bundles — dorsal and two septal — do not reach the pulp tissues.

The exocarp of citrus fruit is the outer colored peel, often referred to as the flavedo (Schneider, 1968) (**Figure 1A**). Proceeding inward is the albedo, the spongy white part of the peel. Most cell layers of the albedo are considered to be mesocarpal tissue, and the two or three innermost cell layers are referred to as endocarp (**Figure 1**). In mandarins, the albedo disintegrates during fruit maturation, leaving only the vascular system (reticula), which gives this group its name, *Citrus reticulata*. The pulp, the edible part of the fruit, is composed of juice sacs/vesicles that develop from the endocarp at an early stage of fruit development (**Figure 1**). Some authors refer to the juice sacs as endocarp, while others consider them to be a separate tissue. The juice sacs develop into the ovary locule, defined as the section in which the ovary wall that develops into fruit. The carpel and the juice sacs are covered by the same epidermal layer of segment epidermis (**Figure 1B**). The juice sac is connected to the wall by a stalk, which joins the segment epidermis, so the latter provides one continuous layer covering both the segment and the juice sac. Three major vascular bundles, a dorsal and two side (septal) bundles, are found in each section. Most juice sacs initiate from the dorsal wall, but some develop from the side wall, adjacent to the side vascular bundle (Koch and Avigne, 1990). When present, seeds develop in the inner side of the fruit, where the carpels merge or along the ovary wall. Nutrition is supplied by a specific bundle, termed seed (or central) bundle, reaching from the fruit pedicle to the center of the fruit.

The juice sac is a unique structure, found only in fruit of the genus *Citrus* and its close relatives. It is often referred to as a “sac of juice,” but this is misleading; the juice sac is composed of various layers of cells, each with distinct morphology (Shomer, 1975; Burns et al., 1992; Burns et al., 1994). The vesicle primordia emerge from the endocarp soon after fertilization and fruit set. In a few cases, juice sac primordia are visible even before fertilization and fruit set, mainly when fertilization does not occur and parthenocarpic fruit develop (this is the case in many commercial citrus cultivars) (Burns et al., 1992). During fruit development, the vacuole of the juice sac cell becomes greatly enlarged, occupying over 90% of the total cell volume, and releases its content as juice. At fruit maturity, the vacuole contains about 100, 75, and 90% of the total cellular sucrose, hexose and citrate, respectively (Echeverria and Valich, 1988).

The juice sac is considered the major fruit sink; however, it is disconnected from the vascular system, which ends in the albedo (**Figure 1B**). This unique characteristic determines photoassimilate translocation rate into the sink cells and therefore, rate of fruit development, and the time required to reach maturity.

## Citrus Fruit Development in Relation to Changes in Sugar and Acid Contents and Climatic Effects

In many citrus cultivars, the major external change that marks the conversion of the citrus ovary into a fruitlet is usually petal fall (Spiegel-Roy and Goldschmidt, 1996). Fruit development is divided into three overlapping stages: cell division (stage I), cell expansion (stage II), and fruit maturation (stage III) (Bain, 1958). During stage I, fruit growth is relatively moderate, and the peel, especially the albedo, thickens by cell division. During this stage, juice sacs grow out *via* cell division into the locule. Stage II is characterized by rapid fruit growth, mostly due to juice cell expansion. During stage III, the rate of fruit volume increase is greatly reduced. Externally, the major change is color break, and internally, sugar and acid levels reach the desired levels for harvesting and consumption, as discussed further by Spiegel-Roy and Goldschmidt (1996). Changes in secondary metabolites give the fruit its unique aroma and flavor (Tadeo et al., 2008). As there is no respiration burst or autocatalytic ethylene production, the citrus fruit does not undergo the classical ripening process, typical of climacteric fruits. For a given citrus cultivar, the final flavor quality of the fruit has to be determined empirically and depends, largely, on consumer preference (Goldenberg et al., 2018). The completion of fruit development is cultivar-dependent, with some cultivars, such as Satsuma mandarin (*Citrus unshiu*), being ready for harvest 5–6 months after flowering, whereas others, such as Valencia orange (*Citrus sinensis*), are harvested 12–14 months after flowering (Ladaniya, 2008). In hot climates, fruit development is accelerated, potentially reducing the time needed for fruit maturation by ca. 50% (Reuther, 1973). Sugar and acid level in the pulp are the two major fruit quality determinants. The major organic acid associated with pulp total acidity is citrate, which begins to accumulate during stage II of fruit development, when the fruit and its juice vesicle cells enlarge rapidly (Hussain et al., 2017). The accumulation continues for a few weeks, reaching a peak when the fruit volume is about 50% of its final value, then the acid declines gradually as the fruit matures. In most varieties, there is a slight increase in sugar content early in fruit development, but the major increase occurs during stage III, when the acid content declines (Sinclair, 1984). In citrus, the major translocated sugar is sucrose and in many varieties, it accumulates to double the level of glucose or fructose (Goldschmidt and Koch, 1996). Maturation index, which determines the fruit’s internal quality, is the ratio between total soluble solids (TSS, BRIX) and total acidity. As the acid content declines toward harvest, sugars account for most of the TSS.

As already noted, climate plays a major role in fruit development and maturation. Most of the commercial citrus cultivars were selected or bred in the subtropical regions of the world, and they are therefore adapted to regions where maturation occurs during the cool season (Wu et al., 2018). In hot climates, such as in the tropics, fruit maturation is accelerated and the major factor affected by temperature is the fruit acid level, with a linear relationship between the accumulation of heat hours and acid decline (Reuther, 1973). Therefore, in hot climates, the fruit reaches its maturation index faster than in colder climates, and tends to be too sweet. However, this is only part of the problem. Citrate catabolism is associated with an increase in alcohols, aldehydes and other secondary metabolites associated with reduced flavor and fruit decay (Porat et al., 2002). Therefore, in hot climates, the time during which the fruit is harvestable and marketable is considerably shortened, and fruit decay occurs faster than in the colder regions (Reuther, 1973). Hot climate has the opposite effect on color break, which requires the correct number of cold night-time to develop (Goldschmidt, 1988; Iglesias et al., 2007; Tadeo et al., 2008). Therefore, not only the fruit decay faster in hot climates, but their color does not fully develop, and in extreme cases may even remain green. One of the expected outcomes of climate change is warmer winter temperatures with shorter cold-night times (Cleland et al., 2007). As most citrus cultivars are harvested during this season, the effect of global warming is expected to be negative on both internal and external citrus fruit quality.

## Photoassimilate Translocation Into Fruit and Sugar Metabolism

### Sink Strength and Its Control by Sucrose Hydrolysis in the Sink

**Figure 2:**
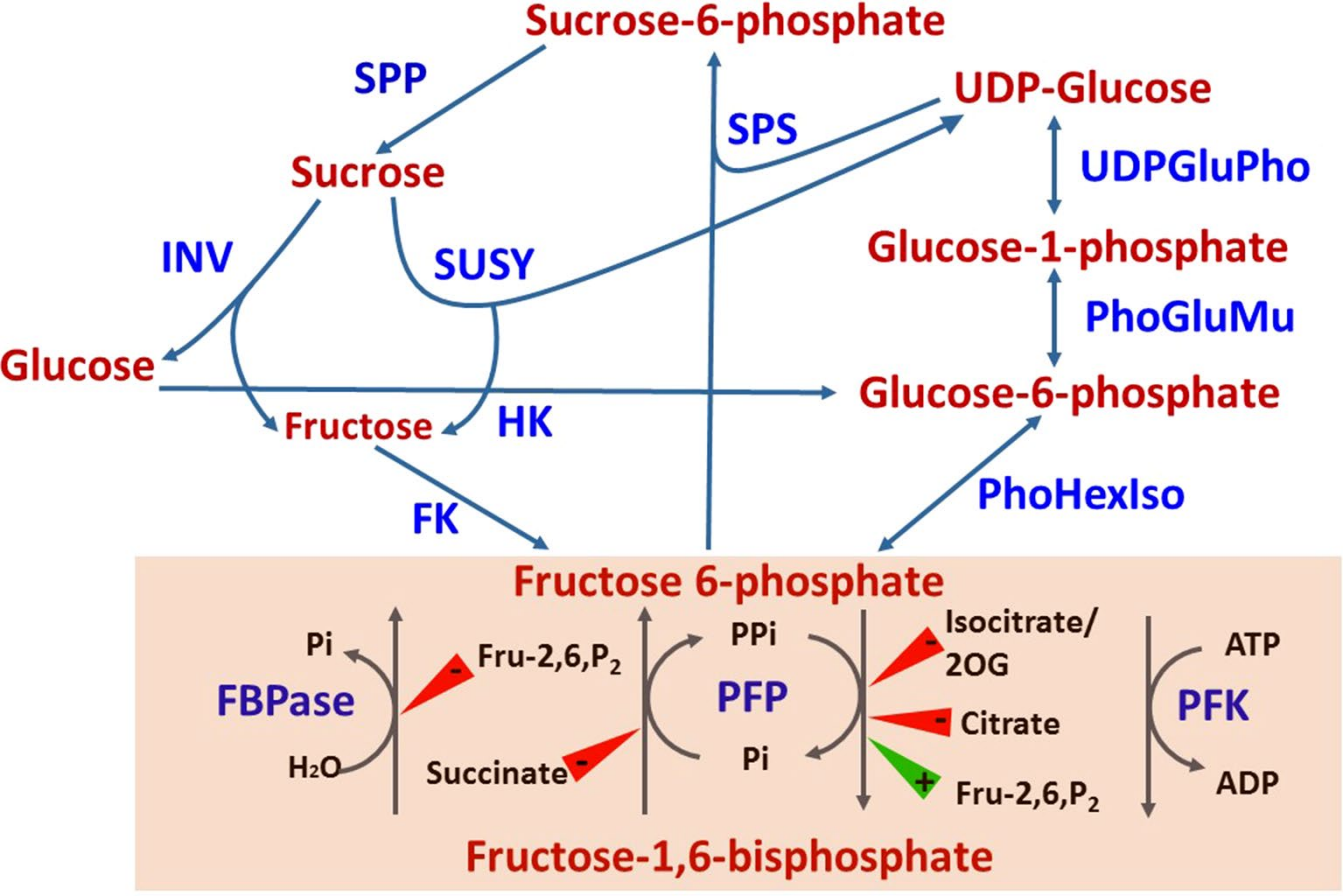
Sugar metabolism and the interconversion of fructose-6-phosphate to fructose-1,6-bisphosphate (shaded). Compound inhibiting (red triangle) or inducing (green triangle) the activities of FBPase, PFP and PFK are shown. INV, invertase; SUSY, sucrose synthase; SPS, sucrose phosphate synthase; SPP, sucrose phosphate phosphatase; HK, hexose kinase; FK, fructokinase; UDPGluPho, UDP glucose phosphatase; PhoGluMu, phosphoglucose mutase; PhHexIso, phosphohexose isomerase; FBPase, fructose bisphosphate phosphatase; PFP, pyrophosphate-dependent fructose 6-phosphate kinase; PFK, ATP-dependent fructose 6-phosphate kinase; Fru-2,6-P_2_, fructose-2,6, bisphosphate; 2OG, 2-oxoglutarate.

Sink strength is determined by the sink’s size and activity (reviewed in Sonnewald et al., 1994; Chang and Zhu, 2017; Smith et al., 2018). In crop plants, it is defined in practice by yield parameters (fruit quantity, fruit size, etc.), and quality parameters, such as carbohydrate (BRIX) and protein levels. Fruit size is genetically controlled, but physiological parameters, such as sink position in relation to other sinks and source tissues, and the time it takes to develop, also affect sink size and therefore, its strength (Bangerth and Ho, 1984; Ross-Ibarra, 2005). In tomato, there are over 30 loci that define fruit size, with many genes acting to control cell division at various developmental stages (Barrero et al., 2006). Practically, sink activity is defined as the rate of photoassimilate translocation and their contribution to growth and developmental processes relative to their accumulation. To simplify the discussion, we will refer here only to sugars, as the major photoassimilates in fruit in general, and in citrus fruit in particular. As with many other plant species, in citrus, sucrose is the major sugar translocated from the leaves to the fruit (Goldschmidt and Koch, 1996). During fruit maturation, it is the major accumulated sugar, with a sucrose:glucose:fructose ratio of 2:1:1 in many cultivars (Komatsu et al., 2002) and references therein). In many cases, sucrose accumulation is detected early in fruit development, indicating a higher translocation than utilization rate (Hiratsuka et al., 2017). As discussed further on, sucrose catabolism into hexoses within the fruit provides the central mechanism controlling sink activity, and therefore sink strength. Sucrose is hydrolyzed either to fructose and UDP-glucose by sucrose synthase (SuSy), a bidirectional enzyme, or to glucose and fructose by invertase, a unidirectional enzyme (**Figure 2**) (Roitsch and Gonzalez, 2004). Following hydrolysis, glucose and fructose are phosphorylated to glucose-6-phosphate and fructose-6-phosphate by hexose kinase and fructokinase, respectively, while UDP-glucose is phosphorylated to glucose-1-phosphate by UDP-glucose phosphorylase. While SuSy is cytosolic, sucrose hydrolysis by invertase is performed in the apoplasm by cell-wall invertases, in the cytosol by neutral/alkaline invertases, and in the vacuole by acidic invertases. The enzyme is modulated post-translationally by invertase inhibitor, which might act *in vivo*, but not necessarily *in vitro* (Roitsch and Gonzalez, 2004; Katz et al., 2007; Palmer et al., 2015). In a few plant systems, it has been shown that alteration of the activities of SuSy and various forms of invertase results in altered yield and/or carbohydrate levels, and thus altered sink strength. For example, one amino acid change in the tomato cell-wall invertase LIN5 enhanced specific activity of the enzyme, the rate of sucrose uptake and, overall, BRIX (Fridman et al., 2004; Baxter et al., 2005). Apoplasmic expression of a yeast invertase gene in potato enhanced tuber size (Sonnewald et al., 1997). Increased expression of cucumber SuSy induced sucrose and starch accumulation and increased fruit size (Fan et al., 2019). Transgenic downregulation of tomato SuSy resulted in reduced sucrose uptake early in fruit development, reduced fruit set, reduced fruit number and reduced fruit size (D’Aoust et al., 1999). Similarly, reduced expression of acid invertase and SuSy in muskmelon and cucumber, respectively, reduced fruit size and sucrose level (Yu et al., 2008; Fan et al., 2019). Phenotypes associated with reduced sink strength were also demonstrated in carrot roots by downregulating vacuolar and cell-wall invertases as well as SuSy (Tang et al., 1999; Tang and Sturm, 1999). Taken together, these studies demonstrated the importance of invertases and SuSy for sink strength and supported the notion that sink strength is controlled, at least in part, within the sink cells and/or at translocation points, i.e., zones of phloem unloading.

### Mechanisms of Phloem Unloading

Sugar transport from the leaf to the collecting phloem is defined as sugar or phloem loading, and its release from the transport system, the releasing phloem, into the sink cell is defined as sugar or phloem unloading (reviewed in Rennie and Turgeon, 2009; Turgeon and Wolf, 2009; Zhang and Turgeon, 2018). Movement of photoassimilates from the leaves to the sink through the stem *via* the transport phloem is a complex process. Although a major driving force is the concentration gradient between source and sink according to the pressure flow hypothesis (Münch, 1930), long-distance movement requires in and out movement of solutes from the transport system to the surrounding tissue, temporal accumulation, and energy investment (Thompson, 2006). The mechanism(s) of sugar unloading has been investigated in a number of fruit and other sink organs, such as tomato, grape berry, cucumber, apple, walnut and potato tuber, by microscopy, fluorescent dyes, immunolocalization of sugar transporters, and use of transporter inhibitors (Ruan and Patrick, 1995; Wu et al., 2004; Zhang et al., 2004; Zhang et al., 2006; Nie et al., 2010; Hu et al., 2011; Palmer et al., 2015; Chen et al., 2017). Unloading is operated by two major mechanisms, symplasmic, in which sugar transport occurs through the plasmodesmata connecting the transport cells and the sink cells, and apoplasmic, where sink cells are not connected symplasmically to the transport cells, and transport must therefore cross membranes through the apoplasm, usually using facilitated transport mechanisms (reviewed in Rennie and Turgeon, 2009; Turgeon and Wolf, 2009; Zhang and Turgeon, 2018). In some fruit, such as cucumber, apple, and kiwifruit, unloading is apoplasmic throughout fruit development (Zhang et al., 2004; Hu et al., 2011; Chen et al., 2017). In other cases, such as tomato fruit and grape berry, there is a shift between symplasmic and apoplasmic unloading (Ruan and Patrick, 1995; Zhang et al., 2006), whereas in the potato tuber, the shift is from apoplasmic to symplasmic (Viola et al., 2001). In jujube, two shifts occur during fruit development; apoplasmic unloading early in fruit development shifts to symplasmic unloading, which then shifts back to apoplasmic unloading close to ripening (Nie et al., 2010). The two types of unloading occur simultaneously during walnut fruit and seed development, symplasmic in the seed and apoplasmic in the fruit (Wu et al., 2004). Overall, symplasmic unloading is considered faster than apoplasmic unloading (Zhang and Turgeon, 2018). Therefore, the mechanism is dependent on the rates of sink development and photoassimilate utilization versus the rate and form of their accumulation, in order to avoid an increase in osmoactive molecules in the cytosol. For instance, in tomato fruit and grape berry, fruit development and sugar utilization are rapid during the first half of their development, requiring symplasmic unloading. Later, when the fruit shifts from a utilizing to accumulating sink, the unloading rate is reduced by shifting it to apoplasmic. During the first half of potato tuber development, it accumulates soluble, osmoactive sugars, and therefore apoplasmic unloading is required, but during later stages, starch (a non-osmoactive compound) is accumulated, allowing a faster unloading. Jujube fruit is characterized by rapid growth during the middle stages of its development, and therefore apoplasmic unloading is interrupted by symplasmic unloading during this stage.

### Sugar Metabolism in Citrus Fruit and Possible Mechanisms of Its Unloading and Movement

**Figure 3:**
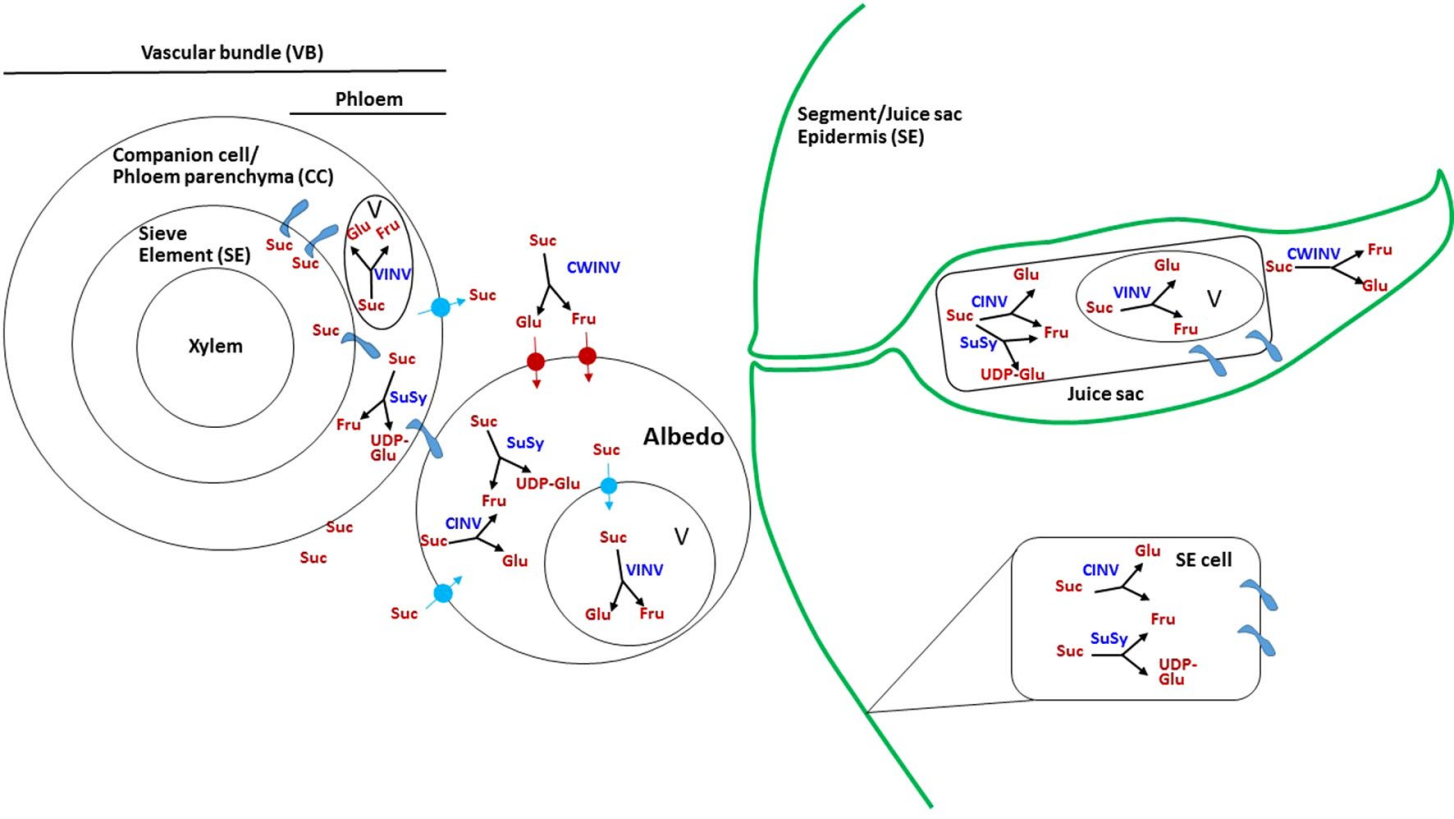
Possible mechanism of sugar transport and metabolism in citrus fruit. Schematic presentation showing the possible paths of sucrose movement and hydrolysis in the various fruit tissues. Suc, sucrose; Glu, glucose; Fru, fructose; UDP-Glu, UDP glucose; V, vacuole; SUSY, sucrose synthase; VINV, vacuolar invertase; CWINV, cell-wall invertase; CINV, cytosolic invertase; 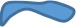 plasmodesmata.

As phloem unloading has never been investigated directly in citrus fruit, the mechanisms in the various fruit tissues are unclear (Goldschmidt and Koch, 1996). Nevertheless, potential mechanisms can be discussed based on the following: (i) the kinetics of sugar transport into the various fruit tissues, vascular bundles, segment epidermis, stalk of the juice sac, and juice sac, as determined using photoassimilate distribution by ^14^CO_2_-feeding of leaves, through either continuous labeling or pulse-chase experiments (Koch, 1984; Koch and Avigne, 1984, Koch and Avigne, 1990; Hiratsuka et al., 2017), (ii) the steady-state distribution of sucrose and hexoses, as well as the activities of sugar-metabolizing enzymes and their protein levels in the various fruit tissues, especially during intensive sugar uptake (Echeverria and Valich, 1988; Lowell et al., 1989; Tomlinson et al., 1991; Echeverria, 1992; Echeverria et al., 1992; Nolte and Koch, 1993a; Nolte and Koch, 1993b; Kubo et al., 2001; Hiratsuka et al., 2017). While the activities of sugar-metabolizing enzymes have been well-studied and characterized, understanding their physiological role during the various stages of fruit development is more challenging. In tomato, for instance, about 20 days post-anthesis, SuSy activity decreased and the activity of an apoplasmic invertase, eventually identified as LIN5, was induced (Yelle et al., 1991; Fridman et al., 2004). This shift was associated with the well-studied shift from symplasmic to apoplasmic unloading and with the conversion of the fruit from utilizing to accumulating sink (Ruan and Patrick, 1995). It might be concluded, therefore, that in tomato fruit, SuSy activity is required to maintain a high rate of sucrose utilization, whereas invertase activity is associated with hexose accumulation. Clearly, the equivalent information is still missing in citrus fruit. In the following, photoassimilate movement and distribution, as well as the activities of sugar-metabolizing enzymes and their protein levels are described for the various fruit tissues (**Figure 3**). In most of the studies, the activities of the various forms of invertases are defined by their pH optima and solubility. Herein, alkaline/neutral-soluble invertase is referred to as cytosolic invertase, acid-soluble invertase as vacuolar invertase, and acid-insoluble invertase as cell-wall invertase (Roitsch and Gonzalez, 2004).

#### Vascular Bundle

^14^C-photoassimilates, mostly as sucrose, were first detected in the dorsal vascular bundle, which seems to be the major transporting bundle, and to a lesser extent in the septal and central vascular bundles as well (**Figure 1**) (Koch, 1984; Koch and Avigne, 1990; Hiratsuka et al., 2017). Using pulse-chase experiments, maximal radiolabel was recovered after 6 h of labeling, but it was remarkably reduced after 24 h. Companion cells, phloem parenchyma and sieve elements are usually connected through plasmodesmata, thus allowing relatively rapid sugar movements. As this movement is slowed down in the albedo and pulp tissues, it can be assumed that temporal storage of sugars occurs in the companion cells/phloem parenchyma. The activity of vacuolar and cytosolic invertases, as well as of SuSy, might well be indicative of such storage. Indeed, SuSy activity was relatively high in the vascular bundle, especially during high sugar translocation, and the protein was strongly immunolabeled in the companion cells (**Figure 3**) (Lowell et al., 1989; Tomlinson et al., 1991; Nolte and Koch, 1993b; Hiratsuka et al., 2017). Acid and soluble invertase activities were also relatively high in the vascular bundle (Lowell et al., 1989; Tomlinson et al., 1991), supporting this notion. Cell-wall invertase was also present in the vascular bundle (Lowell et al., 1989), but this might represent apoplasmic movement from the vascular bundle into the surrounding albedo cells.

#### Albedo Cells

The vascular bundle terminates near the segment epidermis. However, albedo cells are present between the bundle and the segment epidermis, and therefore phloem unloading is expected to occur primarily into albedo cells before sugar reaches the pulp tissue (**Figure 3**) (Tomlinson et al., 1991). In young fruitlets and fruit, the albedo is the major tissue; cell division terminates within 4–5 weeks post-anthesis, and fruit growth during stage II is achieved by pulp expansion (Bain, 1958). Therefore, it might be assumed that most of the sugar in the albedo is transported, while only a minor part of it is required for albedo cell metabolism and development. A considerable amount of sucrose and hexoses are found in albedo cells (Lowell et al., 1989; Hiratsuka et al., 2017). Pulse-chase experiments demonstrated that most of the radiolabel remains in the post-phloem compartment, i.e., albedo cells, for about 24 h before reaching the pulp tissues, juice sac and segment epidermis (Koch and Avigne, 1990). This slowing of sugar movement could indicate that most of the transport is *via* the apoplasmic path. The presence of insoluble acid invertase activity could provide an indication for this type of unloading; such activity has been detected in albedo cells, although at a lower level than in other tissues—the vascular bundle, segment epidermis, and juice sacs (Tomlinson et al., 1991). In addition, the albedo contained considerable activities of SuSy, vacuolar invertase and cytosolic invertase (Lowell et al., 1989; Kubo et al., 2001; Hiratsuka et al., 2017); in fact, the activity of vacuolar invertase was strongest in the albedo compared to other fruit tissues, indicating active storage of sucrose/hexoses in the albedo after unloading. Obviously, the presence of plasmodesmata and a symplasmic pathway between the vascular bundle and albedo cells cannot be ruled out at this stage.

#### Segment Epidermis

As already noted, the segment epidermis provides a continuous layer with the juice sac epidermis (**Figure 1**). It is considered part of the transport tissues, and therefore enzymatic activities are sometimes reported for the vascular bundle and segment epidermis together (Lowell et al., 1989), although in other cases they are separated (Tomlinson et al., 1991; Kubo et al., 2001; Hiratsuka et al., 2017). A considerable percentage, about 30%, of the total radiolabel could be recovered in the epidermis, with maximal accumulation between 24 and 48 h after feeding in a pulse-chase experiment using grapefruit (*Citrus* × *paradisi*) (Koch and Avigne, 1990). With continuous labeling, about 50% of the total radiolabel was recovered in the segment epidermis within 24 h. However, when radiolabeled sugars were quantified in Satsuma mandarin after 48 h of feeding with ^14^CO_2_, the segment epidermis displayed the lowest amount per fresh weight or per fruit (Hiratsuka et al., 2017). These discrepancies could be due to different experimental designs or reflect cultivar differences. Regardless, the segment epidermis provides a strong sink, and movement of photoassimilates from this sink to the juice sac cells cannot be ruled out. Sucrose hydrolysis in the segment epidermis was mediated by relatively high activities of SuSy and soluble invertase (**Figure 3**) (Tomlinson et al., 1991; Kubo et al., 2001; Hiratsuka et al., 2017). The activities of vacuolar and cell-wall invertases were not reported, and it might therefore be assumed that most of the cell-to-cell movement is through the symplastic pathway.

#### Juice Sac Stalk

Photoassimilates were detectable in the stalk of the juice sacs as early as 6 h after ^14^CO_2_ feeding, as found by pulse-chase experiment. However, with continuous exposure, the kinetics of radioactivity accumulation were higher between 24 and 48 h of exposure (Koch and Avigne, 1990). Sugar-metabolizing enzymes were not monitored in the stalk separately from the juice sac, but the same mechanisms are likely to be operating in both parts of the juice sacs.

#### Juice Sacs

As the edible part of the fruit, sugar metabolism and transport in the juice sac have received more attention than in other fruit parts. Photoassimilate transport proceeds to the inner part of the juice sac (**Figure 3**). Following 1 h of ^14^CO_2_ feeding to a source leaf next to grapefruit fruit, and 1 week of translocation, about 60% of the label was found in the juice sacs, with similar results in Satsuma mandarin (Koch and Avigne, 1984; Hiratsuka et al., 2017). A maximal rate of radiolabel accumulation in pulse-chase experiments was reached between 24 and 48 h of labeling (Koch and Avigne, 1990). Movement from the stalk to the distal part of the juice vesicle is relatively slow, and may take up to 96 h in the case of pomelo juice vesicles, which can reach 3 cm in length (Goldschmidt and Koch, 1996). Interestingly, whereas in grapefruit juice sacs, most of the labeled assimilates were recovered as sucrose, in Satsuma mandarin, fructose was predominant (Lowell et al., 1989; Hiratsuka et al., 2017). The accumulation of sucrose per fresh weight peaked in the juice sacs during stage II of fruit development (Lowell et al., 1989). Sucrose hydrolysis seemed to be mediated by all enzymes, as the activity of SuSy and that of the three forms of invertase were detected in the juice sacs (Echeverria and Valich, 1988; Lowell et al., 1989; Echeverria, 1992; Kubo et al., 2001; Hiratsuka et al., 2017). However, most studies showed that the activity of vacuolar invertase was relatively high, followed by SuSy activity. The activity of cell-wall invertase was also detected, but at a lower level, and soluble invertase activity was lowest. The relatively slow sugar transport in the juice sacs suggests diffusion. The presence of plasmodesmata has so far not been demonstrated, and cell-to cell movement might also follow a symplasmic pathway. Considering the relatively high activity of the vacuolar invertase, temporal storage and compartmentalization of sugars should occur during transport. Moreover, as the activity of cell-wall invertase was also demonstrated, apoplasmic movement cannot be ruled out, and it might also play a role in temporal storage. Lowell et al. (1989) indicated that young fruit might behave differently than mature ones, as the former displayed uphill transport in terms of sugar concentration whereas fully grown fruit displayed downhill transport (Lowell et al., 1989). Interestingly, out of the six SuSy genes in the citrus genome, two were induced in juice sacs during development, with one of them induced in the segment epidermis as well, suggesting that SuSy acts in sucrose mobilization within the juice sacs (Komatsu et al., 1999; Islam et al., 2014). As expected, invertase activity in all cellular compartments was reduced toward fruit maturation, in good correlation with the reduction in the invertase transcripts (Lowell et al., 1989; Katz et al., 2011). The activity and transcript levels of sucrose phosphate synthase genes were induced in Satsuma fruit juice sacs toward maturation, in accordance with an increase in sucrose level; however, in grapefruit, enzyme activity was induced from stage I to stage II of fruit development, and decreased toward maturation (Lowell et al., 1989; Komatsu et al., 1996; Komatsu et al., 1999). This might explain the difference in sucrose levels between the two cultivars, as grapefruit accumulates less sucrose than Satsuma mandarin. Sucrose phosphate phosphatase was also induced during later stages of fruit development, suggesting that sucrose accumulation did not result only from translocation from the leaves but also from active synthesis within the juice sac cells (Komatsu et al., 1999; Katz et al., 2011). Nonutilized sucrose is stored in the vacuole and therefore, sucrose transport across the tonoplast might well play a role in regulating its levels within the cell and even its unloading rate. Sucrose and hexose uptake into tonoplast vesicles of sweet lime (*Citrus limetta*) was not induced by ATP, suggesting facilitated diffusion (Echeverria et al., 1992; Echeverria et al., 1997). Inclusion of acid invertase protein in the vesicles induced sucrose uptake, suggesting that sucrose hydrolysis by invertase or chemical acid hydrolysis within the vacuole provided the driving force for its uptake (Echeverria et al., 1992; Echeverria et al., 1997). An endocytic mechanism for sucrose transport across the tonoplast was also suggested (Etxeberria et al., 2005).

## The Interconversion of Fructose-1-Phosphate and Fructose-1,6-Biphosphate, a Central Step Connecting Sugar and Organic Acid Metabolism

While being transported into the fruit, sucrose can undergo metabolism in a few directions. Hexose phosphate synthesis is an important metabolic step, with the reversible conversion of fructose-1-phosphate (Fru-1-P) and fructose-1,6-biphosphate (Fru-1,6-P_2_) providing a link between sugar and organic metabolism *via* glycolysis/gluconeogenesis pathways (**Figure 2**) (Plaxton, 1996; Fernie et al., 2004). The reaction is catalyzed by two independent mechanisms (Uyeda, 1979; Hofer, 1987; Yang et al., 2014). One involves two enzymes, an ATP-dependent phosphofructokinase (PFK) catalyzing the glycolytic conversion of Fru-6-P to Fru-1,6-P_2_, and fructose-1,6-bisphosphatase (FBPase), catalyzing the reverse, gluconeogenic reaction. The other mechanism is composed of one bidirectional enzyme, pyrophosphate-dependent PFK (PFP) composed of two subunits, PFPα and PFPβ (Mertens, 1991; Muchut et al., 2019). Whereas PFK is generally considered ubiquitous, PFP has been described in prokaryotes and lower eukaryotes, including some bacteria, and some protozoan parasites (Bapteste et al., 2003). In addition, it is found in higher plants, where it is expressed in various tissues (Muchut et al., 2019 and references therein). While plants contain both PFP and PFK, bacteria and protozoa appear to have either one or the other, and yeast and animals contain only the latter (Bapteste et al., 2003). PFK is considered the more abundant enzyme, but its activity in plants is less characterized than that of PFP, due to its instability upon purification. PFK is found in both the cytosol and the plastids, whereas PFP is a cytosolic enzyme. Several hypotheses have been raised to explain the role of PFP in plants, including activation during stress (Krook et al., 2000; Fernie et al., 2001; Mutuku and Nose, 2012; Panozzo et al., 2019). Transgenic up/downregulation of PFP in tobacco, potato, and sugarcane resulted in only minor alternations in plant growth and metabolism (Hajirezaei et al., 1994; Paul et al., 1995; Nielsen and Stitt, 2001; Wood et al., 2002a; Wood et al., 2002b; Groenewald and Botha, 2008; Besir and Cuce, 2018). However, reduced expression of PFP in *Arabidopsis* resulted in delayed development, while higher expression resulted in induced development (Lim et al., 2009). Moreover, knockout mutants suggested that PFP is required for adaptation to salt and osmotic stress during germination and seedling growth (Lim et al., 2014). While Fru-2,6-P_2_ is the major PFK activator in microorganisms and animals, in plants it does not activate PFK but rather PFP (Stitt, 1990). Citrate was found to be an inhibitor of PFP activity, especially in the glycolytic direction (Carnal and Black, 1983), and was suggested to affect the affinity of Fru-2,6-P_2_ binding (Van Praag, 1997a; Van Praag et al., 1998).

PFP was detected in the juice sac cells of Valencia orange and grapefruit along with PFK and FBPase (Echeverria and Valich, 1988; Van Praag et al., 1999). While grapefruit PFP was strongly induced by Fru-2,6-P_2_ in the forward reaction, it was barely affected by the activator in the reverse reaction (**Figure 2**) (Van Praag, 1997a; Van Praag, 1997b; Van Praag et al., 1998), as also demonstrated for potato, pineapple and tomato fruit (Van Schaftingen et al., 1982; Kobayashi et al., 1992; Tripodi and Podesta, 1997). It was also shown that citrate, and to some extent other intermediates of the tricarboxylic acid cycle, inhibit the glycolytic reaction of PFP in grapefruit, whereas the gluconeogenic reaction was barely affected (Van Praag, 1997a). Reduction in PFP activity in the ovaries of open versus closed flowers paralleled the reduction in protein levels of the two subunits, suggesting that the enzyme activity was regulated by its protein levels in the ovary (Kapri, 2003). However, more complex relationships were detected in the fruit, demonstrating the involvement of other mechanisms in regulating PFP activity. Recently, the two subunits of citrus PFP were coexpressed and expressed separately in bacteria (Muchut et al., 2019). Monomeric forms of both subunits were able to catalyze phosphorylation of Fru-1-P, but when coexpressed, the heteromeric form generated activity that was two orders of magnitude larger. While the activity of the heteromeric form was induced by Fru-2,6-P_2_, that of the β-monomer was repressed and the activity of the α-monomer was barely affected.

## Citrate Metabolism and Vacuolar PH Homeostasis in Citrus Fruit

Citrate metabolism, transport and accumulation in citrus fruit have been recently reviewed (Hussain et al., 2017). Here they are described only briefly, with an emphasis on the biochemistry and control of transport mechanisms associated with proton and citrate translocation which have been characterized in citrus fruit.

### Pulp Acidity and Citrate Level

**Figure 4:**
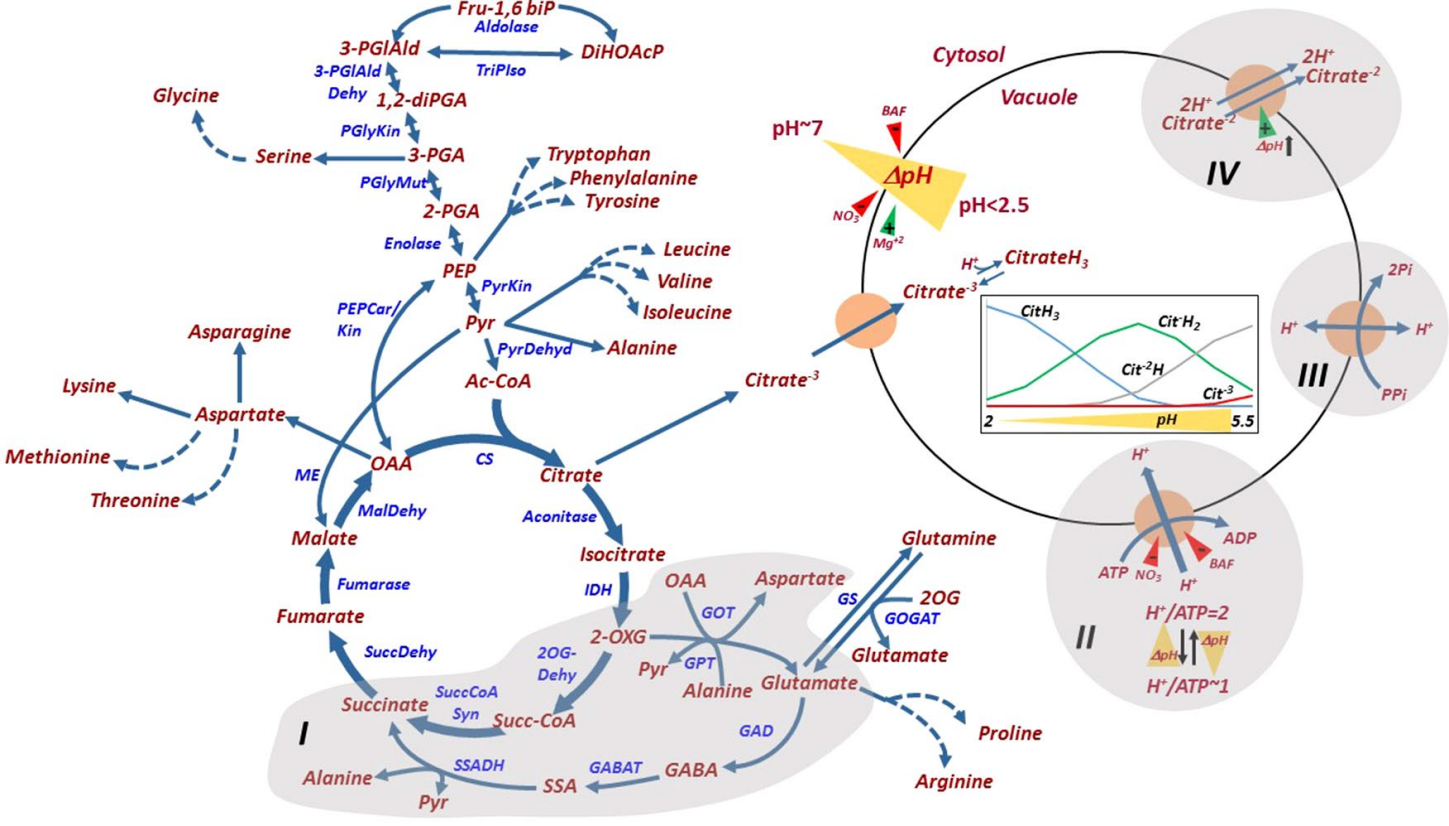
Glycolysis, tricarboxylic acid metabolism, their metabolic connections to amino acids, and citrate and pH homeosthasis in the juice sac cell. The GABA shunt connecting glutamate and 2-oxoglutarate (I), the V-type H^+^-ATPAse (II), the H^+^-pyrophosphatase (III) and the Citrate/H^+^ symporter (IV) are shaded. H^+^/ATP coupling ratio and its pH dependence are indicated. ΔpH (yellow triangle) across the tonoplast is generated by the activities of the H^+^-ATPase and the H^+^-PPiase, and is induced by Mg^+2^ and reduced by BAF and nitrate. V-ATPase is inhibited by bafilomycin (BAF) and nitrate. The Citrate/H^+^ symporter is driven by the acidification of the vacuole (pH decrease). The relative distribution of the various forms of citrate under different vacuolar pH values are shown in the inset figure, as explained in the text. Fru-1,6biP, fructose 1,6-bisphosphatase; DiHOAcP, dihydroxyacetone phosphate; 3-PGIAld, 3-phosphoglyceraldehyde; 1,2-diPGA, 1,2-diphosphoglycerate; 3-PGA, 3-phosphoglyceric acid; 2-PGA, 2-phosphoglyceric acid; PEP, phosphoenolpyruvate; Pyr, pyruvate; Ac-CoA, acetyl-Coenzyme A; 2-OXG, 2-oxoglutarate; Succ-CoA, succinyl-coenzyme A; OAA, oxaloacetate; 2OX, 2-oxoglutarate; GABA, γ‐aminobutyric acid; SSA, succinic semialdehyde; PGlyMut, phosphoglycerate mutase; PyrKin, pyruvate kinase; PyrDehyd, pyruvate Dehydrogenase; CS, citrate synthase; IDH, isocitrate dehydrogenase; 2-OGDehyd, 2-oxoglutarate dehydrogenase; Succ-CoA Syn, succinyl-CoA synthase; SuccDehyd, succinate dehydrogenase; MalDehyd, malate dehydrogenase; PEPCar/Kin, phosphoenolpyruvate carboxykinase; GOT, glucooxaloacetate transaminase; GPT, glucopyruvate transaminase; GOGAT, glutamine oxoglutarate aminotransferase; GS, glutamine Synthetase; GAD, glutamate decarboxylase; GABAT,4-aminobutyrate-2-ketoglutarate transaminase; SSADH, succinic semialdehyde dehydrogenase.

Pulp acidity in citrus fruit is determined by two separate processes, citrate content in the vacuole of the juice sac cell and vacuolar acidification, which can reach 0.3 M and pH 2.0, respectively in lemon and other acidic cultivars (Hussain et al., 2017). Although separate, these two processes are bioenergetically coregulated (Sadka et al., 2000a; Sadka et al., 2000b). During the first half of fruit development, citrate accumulation is accompanied by proton influx which reduces the vacuolar pH. Citrate has three dissociation constants (pKa) — 6.39, 4.77 and 3.14 — and in the vacuole it acts as a buffer by binding protons as they accumulate and reducing the pH, thus providing a driving force for additional proton influx (Müller and Taiz, 2002; Shimada et al., 2006). On the other hand, proton influx provides a driving force for citrate uptake, and probably also for its synthesis. When the vacuolar pH of Navel orange juice sacs was below 3.5, two forms of citrate were detected, citrateH_3_ and citrateH_2_^-^ (**Figure 4**) (Shimada et al., 2006). CitrateH^2−^ and citrateH^3−^ could be detected in pH ≥ 3.5 and pH ≥ 5.0, respectively. During the second half of fruit development, when the acid level declines, citrate removal is accompanied by proton efflux and increasing pH. There is a good correlation among different citrus cultivars between the level of juice pH (representing mostly vacuolar pH) and citric acid concentration (Etienne et al., 2013), and there are no reported cases in which pulp pH and citrate level are both low; therefore, altering citrate concentration will change pH homeostasis, and vice versa. However, early in fruit development, the two processes can be distinguished (Sadka et al., 2000a). Citrate accumulation in Minneola tangelo (*Citrus* × *tangelo*) starts in early June and continues for approximately 3 weeks; during this time, pH is slightly increased, probably due to the dilution effect associated with cell division. Significant pH reduction is only detected after 4 weeks, suggesting that the buildup of some citrate accumulation is required to induce proton influx into the vacuole. This also suggests that citrate accumulation precedes proton accumulation. In other fruit of low and moderate acidity levels, such as melons, i.e., pH 4.5–6.5, some inbred lines with higher pH and higher citrate + malate content than their parents were reported (Burger et al., 2003).

Although citrate is the major organic acid accumulated in citrus fruit, accounting for 90% of the total acids, the synthesis and accumulation of other organic acids have also been reported (Albertini et al., 2006). For instance, in orange, there is a transient increase in quinic and oxalic acids early in fruit development. Malic acid also accumulates to some extent during the maturation of lemon, lime and orange fruit.

### Transport of Citrate and Protons Across the Tonoplast

So far, three mechanisms associated with proton movement across the tonoplast have been identified and characterized in citrus juice sac cells (Müller et al., 1996; Echeverria et al., 1997; Marsh et al., 2000; Shimada et al., 2006): V-type H^+^-ATPase, the major enzyme driving proton influx; H^+^-pyrophosphatase; and citrate/H^+^ symporter, most likely acting to remove citrate^−2^ out of the vacuole along with 2H^+^ (**Figure 4**). Other transport mechanisms, associated with citrate transport across the mitochondrial membrane and citrate movement into the vacuole, have been predicted for other plant species, but not for citrus fruit (Etienne et al., 2013). A P-type ATPase, homologous to the petunia PH5 and PH8, was suggested to play a role in vacuolar hyperacidification (Aprile et al., 2011; Shi et al., 2015). *PH5* and *PH5* were recently shown to be highly expressed in acid cultivars and downregulated in acidless cultivars, due to mutations in the MYB, HLH and/or WRKY transcription factors (Strazzer et al., 2019). While PH5 and PH8 were shown to localize to the vacuole in petunia, their membrane localization and biochemical properties in citrus require further research (Faraco et al., 2014; Verweij et al., 2008).

The identification and characterization of vacuolar transport mechanisms require isolating purified tonoplast vesicles or intact vacuoles (Lin et al., 1977). An array of experimental tools can then be used to study transport across the membranes, such as radiolabeled molecules (citrate), pH-dependent fluorescent dyes such as acridine orange or quinacrine (Stadelmann and Kinzel, 1972). An acidic-inside can be generated in isolated tonoplast vesicles or intact vacuoles through the activation of the V-type H^+^-ATPase or the H^+^-pyrophosphate with Mg–ATP or Mg–PPi and the use of inhibitors (bafilomycin A) or protonophores (gramicidin) to alter the pH gradient. For example, the addition of bafilomycin A inhibits the V-ATPase activity while gramicidin permeabilize the membrane to protons, thus abolishing the DpH across the membrane without affecting the pump hydrolytic activity. Tonoplast vesicles of juice sacs were isolated and purified from acidic cultivars and their acidless counterparts. ^14^C-citrate uptake of acidless pomelo vesicles was about 20% higher than that of acid pomelo, eliminating the possibility that the difference in fruit acidity between these two cultivars was due to citrate transport into the vacuole (Canel et al., 1995). The uptake was enhanced by ATP (**Figure 4**) (Echeverria et al., 1997). Generation of a pH gradient was investigated in tonoplast vesicles of acid (*Citrus aurantifolia*) and acidless lime. As expected, it was induced by Mg–ATP, while bafilomycin and nitrate inhibited ATP hydrolysis and abolished the pH-gradient formation (**Figure 4**) (Brune et al., 2002). Sweet lime tonoplast vesicles appeared to generate a DpH four times faster than those of acid lime, but they had higher H^+^ leakage following H^+^-ATPase inhibition by EDTA than the acid lime, possibly representing their limited *in-vivo* capacity for H^+^ retention. The lemon vacuolar H^+^-ATPase was purified and characterized by Taiz’s group (Müller et al., 1996; Müller et al., 1997; Müller et al., 1999; Müller and Taiz, 2002). They revealed that, in fact, two tonoplast-bound ATPase activities exist, a nitrate-sensitive V-type ATPase that is partially inhibited by vanadate, and a vanadate-sensitive ATPase that is partially inhibited by nitrate (Müller and Taiz, 2002). These results should be taken with caution because of the possible cross-contamination of the tonoplast vesicles with other membrane vesicles. Nitrate inhibition seemed to be dependent on the time of tonoplast vesicle preparation; for the same phenological stage, inhibition peaked during the spring and was minimal during the autumn–winter, suggesting an environmental effect resulting in seasonal changes in membrane lipid composition (Müller et al., 1999). Moreover, the H^+^/ATP coupling ratio varied between 1 to 2 as the DpH increased, displaying a pH-dependent slippage, where the hydrolytic activity and the H^+^ transport are partially uncoupled. Further, the fruit V-ATPase reconstituted into artificial proteoliposomes showed a steeper pH gradient than the corresponding reconstituted epicotyl enzyme (Müller et al., 1997). Overall, the following characteristics seem to allow lemon fruit V-ATPase to generate a steep pH gradient: (i) variable coupling, (ii) low pH-dependent slip rate, (iii) low proton permeability of the membrane, (iv) lower H^+^/ATP stoichiometry, and (v) improved coupling by citrate, the major accumulated organic acid, which also enhance the enzyme’s ability to generate a pH gradient. The pyrophosphatase activity in acid lime fruit was much lower than that of H^+^-ATPase, suggesting the latter as the major mechanism for proton influx (Echeverria et al., 1997). Tonoplast vesicles isolated from juice cells of ‘Valencia’ oranges (*Citrus sinensis* L.) displayed similar V-type ATPase and V-PPiase activities, although a steady-state was reached faster with ATP as substrate. At a DpH of 3 units, V-PPiase synthesized PPi in the presence of Pi, indicating that mature orange juice cells acted as a source of PPi, providing a mechanism for recovery of stored energy in the form of the pH gradient across the vacuole during later stages of development and postharvest storage (Marsh et al., 2000). In summary, in light of the possible presence of an additional tonoplastic H^+^ transport mechanism, P-ATPase, vacuolar proton homeostasis and transport across the tonoplast require further biochemical research.

A vacuolar citrate/H^+^ symporter, CsCit1 (**Figure 4**), homologous to the *Arabidopsis* decarboxylate transporter, was characterized in orange fruit; its mRNA and protein levels coincided with the acid-decline stage, suggesting its role in citrate efflux (Shimada et al., 2006). Yeast cells expressing the *CsCit1* displayed electroneutral coupled citrate–H^+^ cotransport with a stoichiometry of 1citrate/2H^+^.

## Amino Acid Homeostasis in Citrus Fruit

Amino acids have been studied in citrus fruit in relation to the nutritional value of the juice provided the motivation, mostly for early workers, to analyze the levels of free amino acids and their patterns of accumulation during fruit development and storage (reviewed in Sinclair, 1984). The exposure of fruit to stress on-the-tree and cold or heat treatments during storage was associated with the accumulation of several amino acids. Glycolysis and the tricarboxylic acid cycle are metabolically associated to amino acid metabolism (**Figure 4**), its relation to citrate decline and the induction of a γ-aminobutyric acid (GABA) shunt during the second half of fruit development. Moreover, the possible relationships between amino acid accumulation and Huanglongbing (HLB) resistance/tolerance mechanisms have been recently investigated (Killiny and Hijaz, 2016; Killiny et al., 2018; Setamou et al., 2017; Yao et al., 2019).

### Changes in Amino Acid Metabolism During Fruit Development and Upon External Stimuli

In general, all of the amino acids are detected in the juice of mature fruit, with aspartic acid, asparagine, serine, glutamic acid, proline and GABA being the more abundant (reviewed in Sinclair, 1984). A gradual increase in most of the free amino acids was detected during fruit development and toward maturation of Valencia orange (Sinclair, 1984). This increase is associated with citrate decline and it is common to all citrus cultivars (Kimura et al., 2017). However, different trends were detected in Navel oranges (*Citrus* × *sinensis*), with most amino acids and their metabolites decreasing from stage II to III of fruit development (Katz et al., 2011). A comparative analysis of total amino acid contents among various citrus cultivars showed lemon and mandarin with overall higher contents of essential amino acids than pomelo, grapefruit or sweet orange (Wang et al., 2016). Moreover, lemon displayed higher levels of amino acids with bitter taste, such as histidine, phenylalanine and valine, as well as acidic amino acids, aspartic acid and glutamic acid.

Following harvest, citrus fruit are usually subjected to relatively long storage periods at low temperatures. However, heat treatments, which vary from 37°C for 24 h to ∼50°C for a few minutes, prior to storage, are common to reduce pathogenic agents, as well as to induce resistance to chilling and pathogens. The effects of such treatments on amino acid contents and metabolism were investigated, with conflicting results. In Satsuma mandarins, the contents of most amino acids were reduced or remained unchanged following heat treatment and only ornithine showed a consistent increase following the treatment (Yun et al., 2013). On the other hand, Matsumoto and Ikoma (2012) found that most Satsuma mandarin amino acids were heat-responsive, showing a remarkable contents increase during postharvest storage at 20°C or 30°C, but not at 5°C or 10°C. However, two amino acids, ornithine and glutamine, were cold-responsive, suggesting active metabolism during postharvest cold storage.

Changes in amino acid metabolism during fruit development of various cultivars and in the presence of external stimuli have been studied mostly by transcriptomic and metabolomic analyses. The activation of the GABA shunt, a major route for citrate catabolism (**Figure 4**), was identified in a transcriptomic analysis (Cercos et al., 2006) and confirmed by proteomics (Katz et al., 2007); these analyses identified an increase in the transcript of glutamate dehydrogenase, aspartate/alanine aminotransferase, glutamate dehydrogenase, glutamine synthase, GABA amino transferase and succinate semialdehyde dehydrogenase during fruit development, and the presence of their corresponding proteins during the declining-citrate stage of fruit development (Cercos et al., 2006; Katz et al., 2007; Katz et al., 2010; Katz et al., 2011; Lin et al., 2015). Moreover, use of an aconitase inhibitor, which induces citrate accumulation, resulted in induced activities of some of the enzymes of the GABA shunt (Degu et al., 2011). In addition, proteins of most amino acid-synthesis enzymes were induced either from early stage II to stage II or from stage II to stage III of fruit development, including pathways leading to the synthesis of cysteine, glycine, serine, leucine, valine, asparagine, aspartate, alanine, ornithine and glutamine (Katz et al., 2011). Induction of amino acid metabolism was suggested to play a role in the accumulation flavor-associated volatiles (Yu et al., 2015). Comparative transcriptomic analysis of high- and low-citrate oranges showed elevated transcript levels of phenylalanine-, arginine-, proline-, cysteine- and methionine-metabolism genes in the high-citrate orange (Lu et al., 2016). Cold storage of mandarins resulted in major alterations in amino acid metabolism, including the biosynthesis of proline and arginine, and significant enhancement of the catabolism of branched-chain amino acids (Yun et al., 2010; Tietel et al., 2011; Yun et al., 2012). Catabolism of the branched-chain amino acids leucine, isoleucine, and valine releases acetyl-CoA, providing a precursor for amino acid-derived volatiles that are associated with off-flavor development during fruit storage (Tietel et al., 2011). Water stress also induced alterations in the amino acid metabolism suggested to be involved in defense mechanisms against stress (Oliveira et al., 2015).

### Amino Acids and Defense Against HLB

Citrus HLB, caused by the phloem sap-restricted bacterium *Candidatus* Liberibacter, is a serious production threat to the citrus industry in various regions of the world. The bacteria are transmitted by phloem sap-piercing citrus psyllids while they feed, mostly on young expanding vegetative shoots. Different citrus cultivars show varied susceptibility/tolerance to HLB. The differential response seems to be associated with psyllid feeding preferences and with plant tolerance to the bacteria. Based on controlled graft-inoculation experiments, cultivars were classified into three major groups, sensitive, moderately tolerant and tolerant, each showing different symptoms, from severe leaf chlorosis, depressed growth and death in the sensitive cultivars, to fewer and lesser severe symptoms in the tolerant cultivars. The bacteria appeared to be auxotrophic for a few amino acids, supplied by their host. The bacteria were suggested to affect free amino acid availability by altering the expression of amino acid storage proteins, at least in the insect host. To assess whether amino acid metabolism plays a role in the variable citrus tolerance to HLB, metabolomics analyses were performed in various cultivars on healthy and infected trees. Although most of the analyses were performed with phloem sap, and not the fruit, we include their brief description, as some fruit symptoms might also be associated with changes in amino acid metabolism. In a metabolic survey of phloem sap and leaves of citrus cultivars showing varied sensitivity/tolerance to HLB, the levels of all amino acids were elevated in the tolerant cultivars (Killiny and Hijaz, 2016; Killiny et al., 2018). Comparative analyses of amino acid contents in the phloem sap of bacterium-permissive (*Citrus* and psyllid) and non-permissive (non-*Citrus*) hosts showed that seven amino acids, mostly of the glutamate family, were associated with susceptibility, whereas five amino acids, mostly of the serine family, were associated with tolerance/resistance (Setamou et al., 2017). Moreover, high proline-to-glycine ratios were associated with bacterium-permissive hosts. Overall, the level of consistency in these studies in relation to amino acid composition in sensitive/tolerant plant species was not high. HLB-symptomatic Valencia orange fruits showed an overall increase in the level of most detected amino acids as compared to no symptomatic fruit, possibly due to protein degradation (Yao et al., 2019).

## Concluding Remarks

Along with secondary metabolites, products of primary metabolism — carbohydrates, organic acids, amino acids, fatty acids, and their polymeric forms — provide important components to fruit taste, aroma and nutritional value. Fruit vary in their structure, and this variation affects developmental, as well as primary and secondary metabolic processes. The juice sacs — the major pulp component in citrus — are unique among fruit. In this review, we summarize how this unique structure affects photoassimilate translocation, movement, metabolism, and accumulation. Surprisingly, despite intensive research on many aspects of citrus fruit development and metabolism, the mechanisms of photoassimilate unloading have so far not been investigated as in other fruit and sinks, although the research tools are quite well-developed. Here, sugars, organic acids, and amino acids are metabolically connected, and special attention is given to the connecting steps, i.e., the interconversion of Fru-6-P and Fru-1,6-P_2_, and the GABA shunt. In summary, this review attempts to summarize research of primary metabolism in citrus fruit, emphasizing open questions deserving further research.

## Author Contributions

AS and EB wrote the text; LS and IK made the figures and helped with literature search.

## Conflict of Interest

The authors declare that the research was conducted in the absence of any commercial or financial relationships that could be construed as a potential conflict of interest.
